# Semicarbazone EGA Inhibits Uptake of Diphtheria Toxin into Human Cells and Protects Cells from Intoxication

**DOI:** 10.3390/toxins8070221

**Published:** 2016-07-15

**Authors:** Leonie Schnell, Ann-Katrin Mittler, Andrea Mattarei, Domenico Azarnia Tehran, Cesare Montecucco, Holger Barth

**Affiliations:** 1 Institute of Pharmacology and Toxicology, University of Ulm Medical Center, Albert-Einstein-Allee 11, 89081 Ulm, Germany; leonie.schnell@uni-ulm.de (L.S.); ann-katrin.mittler@uni-ulm.de (A.-K.M.); 2 Department of Chemical Sciences, University of Padova, 35121 Padova, Italy; andrea.mattarei@unipd.it; 3 Department of Biomedical Sciences, University of Padova, 35131 Padova, Italy; doazte@gmail.com (D.A.T.); cesare.montecucco@gmail.com (C.M.)

**Keywords:** diphtheria, diphtheria toxin, cellular uptake, membrane transport, EGA

## Abstract

Diphtheria toxin is a single-chain protein toxin that invades human cells by receptor-mediated endocytosis. In acidic endosomes, its translocation domain inserts into endosomal membranes and facilitates the transport of the catalytic domain (DTA) from endosomal lumen into the host cell cytosol. Here, DTA ADP-ribosylates elongation factor 2 inhibits protein synthesis and leads to cell death. The compound 4-bromobenzaldehyde *N*-(2,6-dimethylphenyl)semicarbazone (EGA) has been previously shown to protect cells from various bacterial protein toxins which deliver their enzymatic subunits from acidic endosomes to the cytosol, including *Bacillus anthracis* lethal toxin and the binary clostridial actin ADP-ribosylating toxins C2, iota and *Clostridium difficile* binary toxin (CDT). Here, we demonstrate that EGA also protects human cells from diphtheria toxin by inhibiting the pH-dependent translocation of DTA across cell membranes. The results suggest that EGA might serve for treatment and/or prevention of the severe disease diphtheria.

## 1. Introduction

*Corynebacterium diphtheriae* produces the single-chain diphtheria toxin (DT, 58 kDa), which is the causative agent of diphtheria [1]. DT is efficiently taken up into human cells and its catalytic domain (DTA, 21 kDa) acts as an extremely potent enzyme in the cytosol. DTA covalently transfers ADP-ribose from cellular NAD^+^ onto a modified histidine residue (diphthamide) of the elongation factor 2 (EF-2) thereby inhibiting protein synthesis and causing cell death [2,3], which can be monitored in terms of cell-rounding using HeLa cells [4,5]. DTA is located in the N-terminal domain of DT [6] while the C-terminal part (DTB, 37 kDa) mediates binding of the toxin to susceptible cells and the subsequent transport of DTA into the cytosol. DTB contains a receptor-binding (B) domain, which binds to the heparin-binding epidermal growth factor-like growth factor precursor (HB-EGF) [7,8] and a translocation (T) domain [9], which inserts into the membranes of acidified endosomes [10,11] allowing the membrane translocation of DTA from the endosomal lumen into the cytosol [12,13,14,15,16,17,18]. This process is prevented by bafilomycin A1, an inhibitor of endosomal acidification [19] and can be experimentally mimicked on the surface of cultured cells by exposure of cell-bound DT to an acidic pulse [20]. This triggers the insertion of DTB directly into the plasma membrane and the translocation of DTA into the cytosol where it modifies its substrate [21,22,23].

Translocation of DTA across endosomal membranes is facilitated by host cell factors including the chaperone heat shock protein (Hsp) 90 [24,25] and thioredoxin reductase [5,24,26]. DTA is separated from DTB by cleavage prior or during DT uptake [27] but these two subunits remain linked via an interchain disulfide between Cys-186 of DTA and Cys-201 of DTB [28]. The integrity of the interchain disulfide bond is essential during toxin uptake into endosomes, as well as DTA translocation across the membranes [27,29] but its reduction is necessary for the subsequent release of DTA on the cytosolic side [23] and this process is the rate-limiting step during DT uptake [30]. Reduction of the disulfide bond likely happens after membrane insertion of the T-domain [30] during or after DTA translocation to the cytosol [31]. Thioredoxin 1 reduces this disulfide under acidic conditions in vitro [32] and we recently demonstrated that pharmacological inhibition of thioredoxin reductase prevents DTA transport across cell membranes and protects cells from intoxication [5], implicating that this enzyme is crucial for the reduction of the disulfide bond and the subsequent release of DTA in the cell cytosol of living cells.

The compound 4-bromobenzaldehyde *N*-(2,6-dimethylphenyl)semicarbazone (EGA) was previously identified as a potent inhibitor that protects cells from toxins and viruses which enter the cytosol of cells via acidified vesicles [33,34], such as the *Bacillus anthracis* lethal toxin and DT [34], as well as the binary actin ADP-ribosylating toxins C2 from *Clostridium* (*C.*) *botulinum*, iota from *C. perfringens* and CDT from *C. difficile* [35]. EGA also protects neuronal cells from *C. botulinum* neurotoxins [36] and it was suggested that this compound might modulate intracellular toxin trafficking [34,35,36]. Prompted by these findings, we analyzed the effect of EGA on the intoxication of HeLa cells with DT in more detail. Here, we demonstrate that EGA significantly delays intoxication of cells with DT in a time- and concentration-dependent manner and analyzed the underlying molecular mechanism.

## 2. Results and Discussion

**Figure 1:**
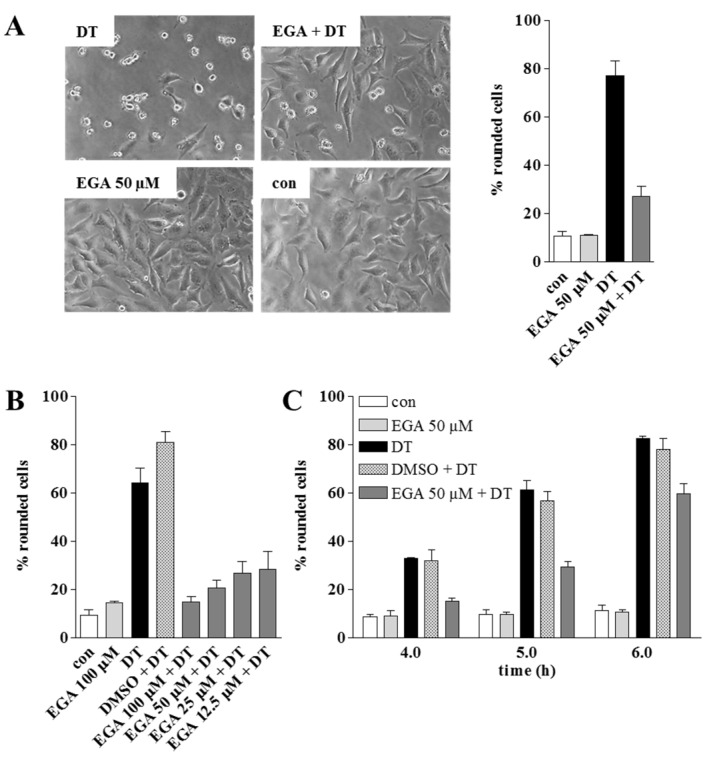
EGA protects HeLa cells from intoxication with DT. Cells were pre-incubated for 1 h at 37 °C with EGA or the solvent DMSO for control. Thereafter, DT (0.86 nM) was added and cells were further incubated at 37 °C. For control, cells were left untreated (con) or incubated with DT in the absence of EGA (DT). At the indicated time points, phase contrast pictures were taken and for quantitative analysis, the percentage of rounded cells was determined. Values are gives as mean ± SD (*n* = 3). (**A**) Protective effect of 50 µM EGA on the intoxication of cells with DT. Representative pictures after 4 h are shown; (**B**) Concentration-dependent inhibition of DT-intoxication after 4 h by EGA, determined in a separate experiment. The maximum dosage of EGA (100 µM) was tested on cells without DT to exclude any morphological alteration of the cells in response to EGA alone. In addition, a DMSO solvent control of the maximum dosage was performed; (**C**) Time-dependent inhibitory effect mediated by 50 µM EGA, determined in a separate experiment with confluent cells.

EGA protects HeLa cells from intoxication with DT. In a first set of experiments the possible inhibitory effect of EGA on the intoxication of HeLa cells by DT was investigated. To this end, cells were pre-incubated for 1 h with increasing concentrations of EGA and then challenged with DT. After different incubation periods, the number of round cells was determined because this is an established, highly specific and sensitive endpoint to monitor the intoxication process [5]. As shown in Figure 1, EGA significantly delayed the DT-induced cell-rounding in a time- and concentration-dependent manner, indicating that EGA interferes with the mode of action of DT in these cells. EGA delayed intoxication with DT even when cells were not grown to confluence and therefore more susceptible to DT. Importantly, EGA alone had no effects on the cells under such conditions (Figure 1A). Adverse effects on the cells were observed at concentrations of 500 µM EGA and above (not shown). The usage of 100 µM instead of 50 µM was still tolerated by the cells but did not result in a relevant improvement of the inhibitory effect (Figure 1B). Therefore, for the following investigation of the underlying molecular mechanism of the EGA-mediated protection of cells from DT, an EGA concentration of 50 µM was used. A prolonged pre-incubation period with EGA (up to 6 h) did not significantly enhance the inhibitory effect of EGA towards DT compared to an 1 h pre-incubation period. Moreover, EGA showed some protective effect towards DT, even when applied 15 min after DT (not shown).

EGA did not completely inhibit intoxication with DT but delayed it, which was expected from our earlier results with EGA and binary clostridial toxins [35] and with DT and other pharmacological inhibitors of toxin uptake [5]. The reason is most likely related to the extreme potency of AB-toxins in cells, as few molecules of their catalytic moieties in the host cell cytosol is usually sufficient to exhibit the full cytotoxic effects [37]. A pharmacological inhibition might therefore not be able to completely prevent the uptake of some DTA molecules into the cytosol over longer incubation periods.

**Figure 2:**
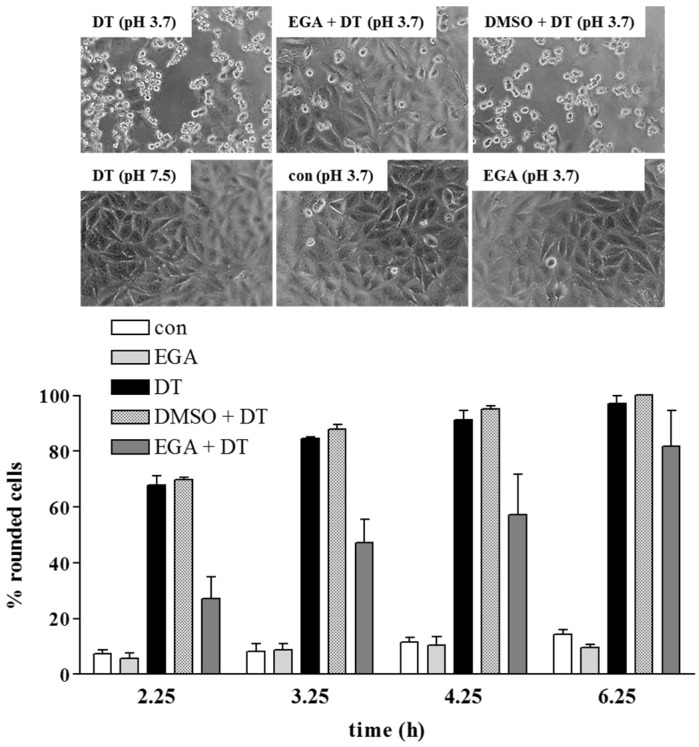
EGA inhibits the pH-dependent transport of the enzyme component DTA of DT across the cytoplasmic membranes in living cells. HeLa cells were pre-incubated for 1 h at 37 °C in serum-free medium with 100 nM bafilomycin (Baf) A1. In addition, some cells were also treated with 50 µM EGA or the corresponding solvent DMSO for control. Subsequently, cells were kept on ice for 10 min. Thereafter, nicked DT (13 nM) was added and cells were incubated for 40 min at 4 °C to enable toxin-binding to the receptors on the cell surface. Cells were further incubated for 15 min at 37 °C and pH 3.7 to trigger the pH-driven membrane transport of DTA. For control (con), cells were incubated at pH 7.5 (not shown) or at pH 3.5 (shown). During this step, there was no EGA present in the medium. Thereupon, all cells were further incubated at 37 °C in neutral medium (pH 7.5) containing serum, Baf A1 ± EGA. At the indicated time points, phase contrast pictures were taken and for quantitative analysis, the percentage of rounded cells was determined. Representative pictures are shown after 3.25 h. Values are gives as mean ± SD (*n* = 3).

**Figure 3:**
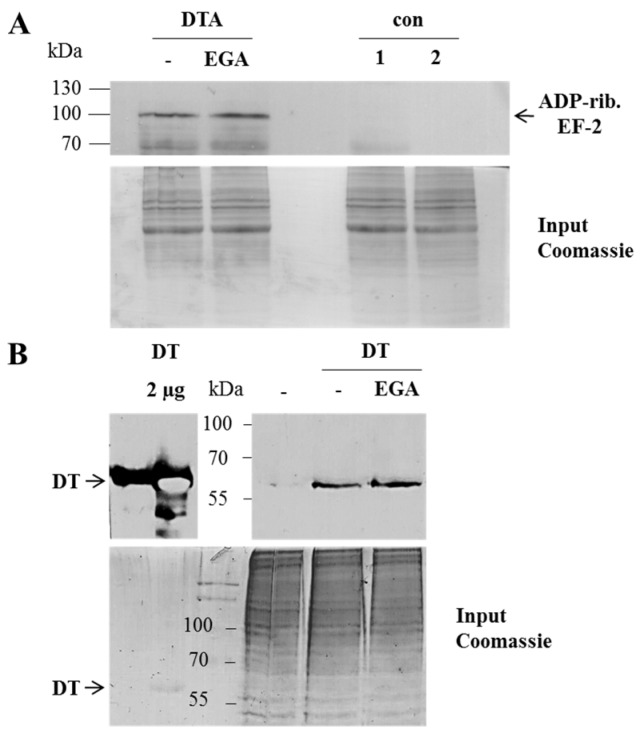

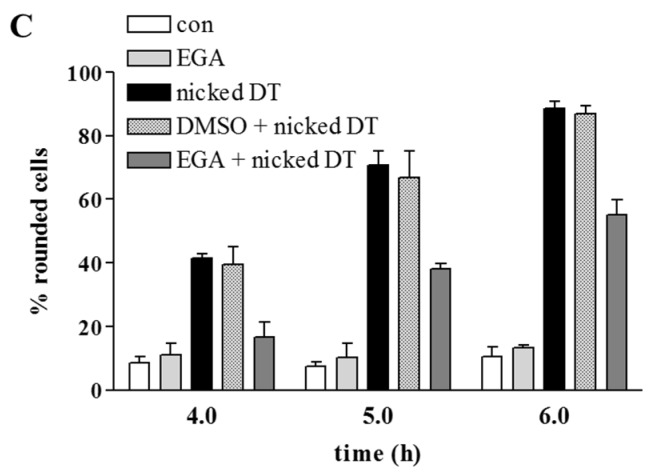
Treatment with EGA has no effect on the in vitro enzymatic activity of DTA nor on the receptor-binding of DT to cells. (**A**) DTA (100 ng) was pre-incubated with 50 µM EGA for 10 min at 37 °C. For control (con), a sample of DTA was left untreated. Thereupon, HeLa lysate protein (20 µg) as well as biotin-labelled NAD^+^ were added followed by an incubation of the samples for 10 min at 37 °C. Thereafter, samples were subjected to SDS-PAGE, blotted and biotinylated (i.e., ADP-ribosylated, indicated as ADP-rib.) EF-2 was detected (upper panel). A sample of HeLa lysate with biotin-labelled NAD^+^ (1) and one of only HeLa lysate (2) were additionally analyzed. As control of equal protein loading, the SDS-gel was stained with Coomassie after the blotting process (lower panel). Noteworthy, there was no obvious difference in the amount of ADP-ribosylated EF-2 when lysate from EGA-treated cells was used (not shown), indicating that EGA-treatment does not interfere with the generation of the diphthamide in EF-2; (**B**) HeLa cells were pre-treated with EGA (50 µM) for 1 h at 37 °C in serum-free medium or left untreated for control. Thereupon, cells were kept on ice for 10 min. Then, DT (34.4 nM) was added followed by further incubation for 1 h 10 min at 4 °C to enable binding of DT to the cell surface. Thereafter, three washing steps with ice-cold PBS were performed to remove unbound toxin and cells were scraped off in 2.5-fold SDS sample buffer containing 10% DTT. Proteins were separated by SDS-PAGE, blotted and bound DT was detected using a specific primary and a peroxidase-coupled secondary antibody and the ECL system (upper panel). For loading control, two dilutions of DT were analyzed on the left side of the same blot. Comparable amounts of total protein were confirmed by Coomassie staining of the proteins in the SDS-gel after the blotting process (lower panel); (**C**) Cells were kept on ice for 10 min before addition of nicked DT (3.4 nM) and further incubation at 4 °C for 30 min. Thereafter, the medium was removed and warm, serum-free medium containing EGA (50 µM) or DMSO was added followed by further incubation at 37 °C. For control (con), cells were left untreated or treated only with EGA without toxin. At the indicated time points, phase contrast pictures were taken and for quantitative analysis, the percentage of rounded cells was determined. Values are gives as mean ± SD (*n* = 3).

EGA inhibits the membrane transport of DTA in cells. The effect of EGA on the membrane transport of DTA was investigated by using a well-established assay where the endosomes acidic environment is mimicked on the surface of cultured HeLa cells. DT was allowed to bind to the receptor at 4 °C where no endocytosis occurs and the normal toxin uptake via acidic endosomes was inhibited by bafilomycin A1 treatment. When such cells are exposed to warm acidic medium, DTB inserts into the plasma membrane and mediates the translocation of DTA into the cytosol where it ADP-ribosylates EF-2 leading to cell-rounding, as shown in Figure 2. No cell-rounding was observed under neutral conditions, by incubation of cells in the acidic medium in the absence of DT, or in the presence of EGA without DT (Figure 2), indicating that cell-rounding is indeed specifically induced by the action of DTA, as described earlier [5,20]. Importantly, the DT-induced cell-rounding was significantly reduced when cells were pre-treated with EGA (Figure 2). Given that this inhibitor did not inhibit the in vitro enzymatic activity of DTA (Figure 3A) nor the binding of DT to HeLa cells (Figure 3B) and it leads to inhibition of intoxication also when applied after receptor-binding of nicked DT (i.e., DT that was proteolytically activated in vitro prior to its application to the cells) (Figure 3C), our results strongly suggest that EGA interferes with the toxin pH-dependent transport. In addition, the results obtained by performing the translocation assay with nicked DT also exclude an effect of EGA on the proteolytic activation of DT during cellular uptake on the cell surface and/or in endosomal vesicles. To demonstrate that the observed inhibitory effect of EGA was mediated by the molecule itself, in each experiment a vehicle control (DMSO) was performed in parallel to EGA treatment. In contrast to EGA, DMSO did not inhibit the intoxication of cells with DT (see Figure 1B,C) or the translocation of cell-bound DT (Figure 2).

## 3. Conclusions

In conclusion, our results demonstrate that EGA protects cultured human cells from intoxication with DT because it interferes with the uptake of DTA into the host cell cytosol. Moreover, our findings suggest that EGA inhibits the pH-dependent transport of DTA across membranes into the cytosol. This step was also inhibited by EGA in the case of clostridial binary toxins, which also deliver their A subunit from acidic endosomes to the cytosol [35]. All toxins which were inhibited by EGA, exploit acidic endosomal vesicles for cellular trafficking, suggesting a common inhibitory mode of action for EGA in cells. The binary anthrax toxins and the binary clostridial actin ADP-ribosylating toxins share an overall comparable mechanism to deliver their enzymatically active components through pores formed by their separate heptameric transport components across endosomal membranes into the cytosol. Thus, a common mechanism by which EGA inhibits the membrane transport of these binary toxins seems plausible. However, EGA inhibits the membrane transport of DT, as shown in this present study, but it did not inhibit the translocation of botulinum neurotoxins across the plasma membrane [36], suggesting that the protective effect of EGA towards both single-chain toxins might not be mediated via the same molecular mechanism. Thus far, the molecular mechanisms by which EGA interferes with the membrane transport of DT are not known and it can be speculated that EGA might block the transmembrane pore formed by the translocation domain of DT or might inhibit host cell factors which are involved in translocation of DTA across endosomal membranes.

However, although the precise mode of action how EGA inhibits the transport of the enzymatically active moieties of bacterial toxins into the cytosol requires further elucidation, this compound inhibits a series of the most powerful and medically most relevant bacterial toxins including DT, botulinum neurotoxins and anthrax toxin, and thus might have very attractive clinical applications. In previous studies [36] the treatment of mice with EGA by multiple intraperitoneally injections were well tolerated. It should be taken into account that higher concentrations of DT might be used in the experimental approach compared to the situation in patients. Moreover, EGA could represent a lead compound and chemical modifications may result in even more efficient compounds for future clinical applications.

## 4. Experimental Section

### 4.1. Materials and Reagents

Cell culture media (MEM) and fetal calf serum (FCS) were obtained from Invitrogen (Karlsruhe, Germany) and cell culture materials from TPP (Trasadingen, Switzerland). The protein molecular weight marker Page Ruler prestained Protein ladder^®^ was purchased from Thermo Fisher Scientific Inc. (Waltham, MA, USA). Complete^®^ protease inhibitor was supplied by Roche (Mannheim, Germany). Biotinylated NAD^+^ was from R&D Systems GmbH (Wiesbaden-Nordenstadt, Germany). Bafilomycin (Baf) A1 was purchased from Calbiochem (Bad Soden, Germany), 2-(4-bromobenzylidene)-*N*-(2,6-dimethylphenyl)hydrazinecarboxamide (EGA) was synthesized as described [36]. The quality of EGA was tested by HPLC-MS analysis. The purity is >95% [36].

Streptavidin-peroxidase was obtained from Roche (Mannheim, Germany), the enhanced chemiluminescence (ECL) system from Millipore (Schwalbach, Germany) and the nitrocellulose blotting membrane from Whatman^®^ (Dassel, Germany). Diphtheria toxin antibody was obtained from GeneTex Inc. (Irvine, CA, USA) and goat anti-mouse IgG-HRP secondary antibody from Santa-Cruz Biotechnology Inc. (Heidelberg, Germany). Diphtheria toxin (DT) was supplied by Calbiochem (Bad Soden, Germany). DT was proteolytically activated in vitro with trypsin to yield nicked DT as described in Section 4.4. and DTA was expressed and purified as described earlier [5].

### 4.2. Cell Culture and Cytotoxicity Assays

HeLa cells were cultivated at 37 °C and 5% CO_2_ in MEM containing 10% heat-inactivated FCS, 1 mM sodium-pyruvate, 2 mM l-glutamine, 0.1 mM non-essential amino acids and 1% penicillin-streptomycin. Cells were trypsinized and reseeded for at most 30 times.

For cytotoxicity experiments, cells were grown in 96-well plates and pre-incubated with EGA or the vehicle DMSO in serum-free medium for 1 h at 37 °C. For control, cells were incubated without inhibitor. Thereafter, DT (0.86 nM) was added and cells were further incubated with toxin plus inhibitor at 37 °C. For analysis of the cellular effect of EGA when added after DT-binding to cells, cells grown in 96-well plates were kept on ice for 10 min in serum-free medium before addition of nicked DT (3.4 nM). After incubation for 30 min at 4 °C, medium was removed and warm, serum-free medium containing EGA (50 µM) or the vehicle DMSO was added followed by further incubation at 37 °C. After the indicated incubation periods, a Zeiss Axiovert 40 CFL microscope (Oberkochen, Germany) with a Jenoptik progress C10 CCD camera (Jena, Germany) was used to visualize the cells to analyze the DT-induced morphological changes. The characteristic rounding up of the cells was taken as specific endpoint to monitor the intoxication process. For quantitative analysis, cells were counted per picture (ImageJ software; NIH, Bethesda, MD, USA) and the amount of rounded cells was determined in percent.

### 4.3. SDS-PAGE and Western Blotting

For immunoblot analysis, according to the method of Laemmli [38], equal amounts of protein were subjected to SDS-PAGE. Afterwards, the proteins were transferred to a nitrocellulose membrane which was then blocked for 1 h at RT with 5% dry milk powder in PBS containing 0.1% Tween-20 (PBS-T) or alternatively overnight at 4 °C. For detection of the biotin-labelled EF-2 or DT, the samples were either probed with streptavidin-peroxidase or a specific antibody against DTA, respectively, for 1 h followed by washing steps with PBS-T. Subsequently, in case of DT, the membrane was probed with anti-mouse secondary antibody coupled to horseradish peroxidase for 1 h followed by further washing steps with PBS-T. Thereafter, the proteins were visualized using the ECL system according to the manufacturer’s instructions. Ponceau S staining of the membrane and Coomassie staining of the gel were used to confirm equal amounts of protein.

### 4.4. Proteolytic Activation (Nicking) of DT

To activate DT, the toxin was treated with trypsin (3 µg/mL) for 3 h at 37 °C and then kept on ice. Following this, trypsin was neutralized by incubation with trypsin inhibitor (30 µg/mL) for 80 min at 4 °C and the concentration of nicked DT was determined using SDS-PAGE.

### 4.5. ADP-Ribosylation of EF-2 by DTA in a Cell-Free System

DTA (100 ng) was pre-incubated for 10 min at 37 °C with EGA (50 µM) or left untreated for control followed by the addition of HeLa lysate protein (20 µg) and biotin-labelled NAD^+^ (10 µM). After further incubation of the samples for 10 min at 37 °C, the proteins were subjected to SDS-PAGE, blotted onto a nitrocellulose membrane. Finally, biotin-labelled (i.e., ADP-ribosylated) EF-2 was detected using Western blotting.

### 4.6. Binding of DT to Its Cell Surface Receptor

HeLa cells were pre-incubated in serum-free medium with EGA (50 µM) for 1 h at 37 °C. For control, cells were left untreated. Following this, cells were kept at 4 °C for 10 min. Then, DT (34.4 nM) was added and cells were further incubated at 4 °C for 1 h and 10 min to enable binding of DT to its specific cell surface receptor. Three washing steps with ice-cold PBS were used to remove unbound toxin and cells were subsequently scraped in 2.5-fold concentrated sample buffer containing 10% DTT [38]. SDS-PAGE was used for protein separation and after blotting, bound DT was detected using a specific antibody against DT and peroxidase-coupled secondary anti-mouse antibody.

### 4.7. DTA Translocation Assay across the Cytoplasmic Membrane of Living Cells

The pH-dependent translocation of DT across endosomal membranes was experimentally mimicked on the cytoplasmic membranes of intact cells and performed as described earlier [5]. In brief, HeLa cells were pre-incubated in serum-free medium with Baf A1 (100 nM) plus EGA (50 µM) or the vehicle DMSO for 1 h at 37 °C. Then, the cells were kept on ice for 10 min followed by an incubation with nicked DT (13 nM) for 40 min at 4 °C to allow binding of the toxin to the cell surface. Thereafter, to trigger pH-driven toxin-translocation across the surface membrane, cells were exposed to an acidic pulse (pH 3.7) for 15 min at 37 °C. Additional wells with toxin-treated cells were treated with neutral medium (pH 7.5) for control. Subsequently, all cells were further incubated at 37 °C in neutral medium containing FCS, Baf A1 (100 nM) ± EGA (50 µM). After the given incubation periods, DT-induced cell-rounding was monitored using photography. From these, the amount of rounded cells was determined in per cent of total cell count per picture.

### 4.8. Reproducibility of the Experiments

All experiments were independently performed at least two times and in the figures, results from representative experiments are shown. Quantification was performed by calculating the values (*n* = 3; *n* = 3 refers to three fields of view in one experiment and several independent experiments were performed, all with comparable results) as the means ± standard deviation (S.D.) using the Prism4 Software (Version 4.0, GraphPad Software Inc., La Jolla, CA, USA, 2003).
